# First report of the influence of temperature on the bionomics and population dynamics of *Aedes koreicus*, a new invasive alien species in Europe

**DOI:** 10.1186/s13071-019-3772-5

**Published:** 2019-11-06

**Authors:** Giovanni Marini, Daniele Arnoldi, Frederic Baldacchino, Gioia Capelli, Giorgio Guzzetta, Stefano Merler, Fabrizio Montarsi, Annapaola Rizzoli, Roberto Rosà

**Affiliations:** 1 Department of Biodiversity and Molecular Ecology, Research and Innovation Centre, Fondazione Edmund Mach, San Michele all’Adige, Trento, Italy; 2 Epilab-JRU, FEM-FBK Joint Research Unit, Province of Trento, Italy; 3 Direction départementale de la protection des population du Nord, Lille, France; 4 Laboratory of Parasitology, Istituto Zooprofilattico Sperimentale delle Venezie, Legnaro, PD Italy; 5 Center for Information Technology, Bruno Kessler Foundation, Trento, Italy; 6 Center Agriculture Food Environment, University of Trento, San Michele all’Adige, Trento Italy

**Keywords:** Mosquito bionomics, Mosquito dynamics, Mathematical model, Vector abundance, Invasive species

## Abstract

**Background:**

*Aedes koreicus* was detected in northern Italy for the first time in 2011, and it is now well established in several areas as a new invasive mosquito species. Data regarding the influence of temperature on mosquito survival and development are not available yet for this species.

**Methods:**

We experimentally investigated the influence of different constant rearing temperatures (between 4 and 33 °C) on the survival rates and developmental times of different life stages of *Ae. koreicus* under laboratory conditions. The resulting data were subsequently used to inform a mathematical model reproducing the *Ae. koreicus* life-cycle calibrated to counts of adult females captured in the field in the autonomous province of Trento (northern Italy) between 2016 and 2018.

**Results:**

We found that temperatures above 28 °C are not optimal for the survival of pupae and adults, whereas temperate conditions of 23–28 °C seem to be very favorable, explaining the recent success of *Ae. koreicus* at establishing into new specific areas. Our results indicate that *Ae. koreicus* is less adapted to local climatic conditions compared to *Ae. albopictus*, another invasive species which has been invading the area for the last three decades. Warmer seasons, which are more likely to occur in the future because of climate change, might extend the breeding time and therefore increase the abundance of *Ae. koreicus* in the study region.

**Conclusions:**

Our findings provide, to our knowledge, the first evidence on how temperature influences the bionomics and dynamics of *Ae. koreicus* and highlight the need for further studies on the phenology of this species in temperate areas of Europe.
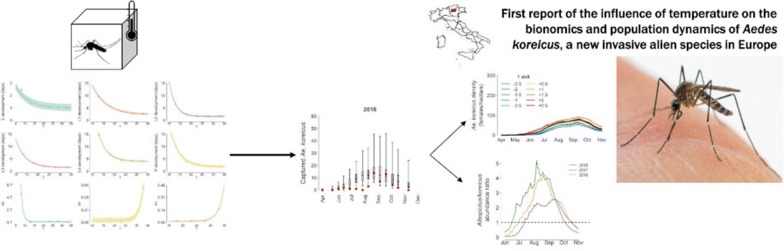

## Background

Several invasion events by alien mosquito species have occurred in Europe in recent decades [1]. The most successful invasive *Aedes* mosquito species is *Ae. albopictus* (Skuse, 1894), which has been recorded in Italy since the end of the 20th century [2]. In 2008, a new aedine mosquito species, *Ae. koreicus* (Edwards, 1917), was found in Europe (in Belgium) for the first time [3]. It was subsequently detected in Italy in 2011 [4], where it has now established in several areas in the northern part of the country, including Veneto region and the autonomous province of Trento, overlapping with the current distribution of *Ae. albopictus* [5, 6]. Habitat-suitability models predict that parts of the main rivers (Po and Adige) valleys will be colonized over the next decade and that *Ae. koreicus* may establish in areas 400–1500 m above sea level, i.e. above the altitude range of *Ae. albopictus* [7]. Thus, a wider geographical range could be colonized by *Aedes* mosquitoes, enlarging the areas with possible *Aedes*-borne disease transmission. In fact, this new invasive species has a potential impact on human and animal health since *Ae. koreicus* can transmit *Dirofilaria immitis*, a heartworm, endemic in northern Italy [8], and could be involved in chikungunya virus transmission [9], whose last European autochthonous outbreak occurred in Lazio region (Italy) in 2017 [10].

Mosquito survival and development are critically affected by environmental temperatures as well (e.g. [11–14]), and quantitative information on this relationship is critical to assess the population dynamics and potential environmental suitability of different mosquito species. Temperature can also affect virus transmission, for example by decreasing the length of the extrinsic incubation period [15] and by increasing transmission probability [16]. To the best of our knowledge, no quantitative data are available for *Ae. koreicus*. To fill this gap, we carried out laboratory experiments to evaluate whether different rearing temperatures exert an effect on the phenology of this species, in particular on the developmental time and survival of immature stages and on adult survival rates and length of the gonotrophic cycle of *Ae. koreicus*.

We designed a density-dependent mechanistic model that incorporates the effect of temperature on the temporal variations of *Ae. koreicus* population for both adults and immature stages. We calibrated our model on the number of trapped adult female mosquitoes recorded in four sites in the province of Trento between 2016 and 2018. This kind of model includes the main mosquito life-cycle processes (from egg hatching to adult mortality), thus replicating closely the actual population dynamics and providing a suitable framework to investigate the main determinants of observed dynamical patterns [17]. Several models have been proposed to explore *Aedes* mosquitoes population dynamics, especially for *Ae. albopictus* [18–20], *Ae. aegypti* (Linnaeus, 1762) [21, 22] and more recently also for *Ae. japonicus japonicus* (Theobald, 1901) [23]. To the best of our knowledge, this is the first attempt to model the population dynamics of *Ae. koreicus*.

The calibrated model was used to forecast possible changes in *Ae. koreicus* seasonal dynamics under alternative temperature scenarios, and to compare the adaptation to the local habitat conditions of *Ae. koreicus* with respect to *Ae. albopictus*, which has been present in the study area for many years [24].

## Methods

### Study area and entomological data

**Fig. 1:**
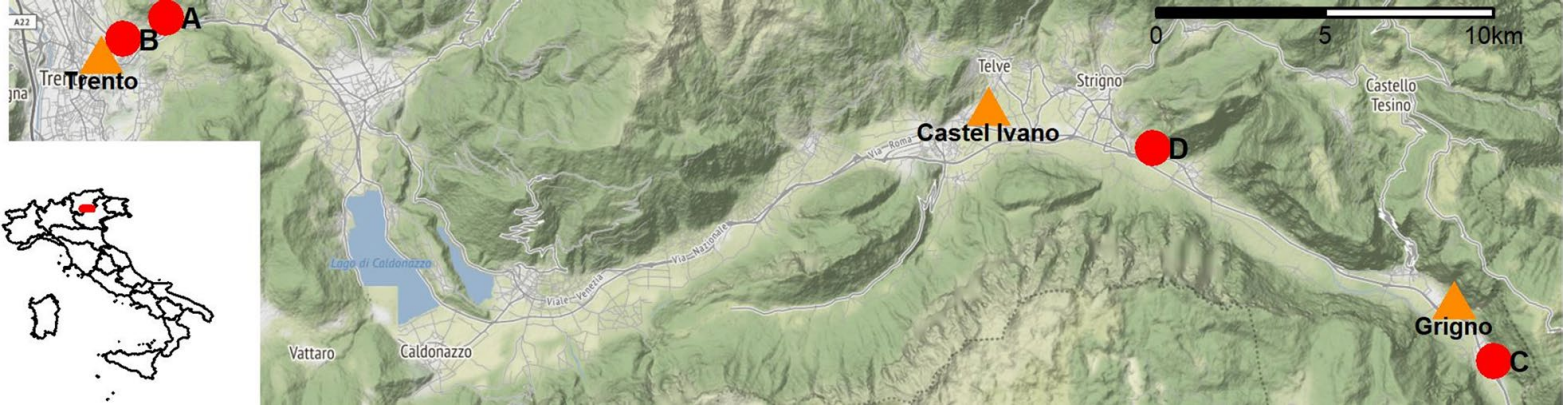
Study area. *Key*: red dots, trap locations; orange triangles, weather stations. Map data © OpenStreetMap contributors [40]

Mosquito sampling was carried out in the province of Trento (46°04′00″N, 11°07′00″E), northern Italy. This mountainous area covers partially the Dolomites and the southern Alps. The climate of the study region is temperate-oceanic with three main areas: subcontinental (the main valleys with more severe winters), continental (the alpine valleys) and alpine (the areas above the tree line) [25]. Four trapping locations (A: 46°05′0.56″N, 11°09′38.27″E; B: 46°04′40.08″N, 11°08′39.01″E; C: 45°59′30.77″N, 11°40′19.34″E; and D: 46°02′55.21″N, 11°32′26.99″E; see Fig. 1) were chosen within three municipalities (Trento, Grigno and Castel Ivano). We deployed one BG Sentinel (Biogents AG, Regensburg, Germany) trap (version 1) for each location. Traps were sheltered from rain and direct sunlight and were baited with BG-lure and CO_2_. They ran for 24 h fortnightly from the end of April or beginning of May to the end of October or beginning of November between 2016 and 2018. Each trap was powered by a 12 V battery. All mosquitoes were collected in a catch bag and carried to Edmund Mach Foundation laboratories (San Michele all’Adige, Italy) for identification under a stereoscope to the species level using taxonomic keys [26, 27].

### Climatic data

Temperature time series (daily average), collected by three ground stations (Fig. 1, orange triangles), were obtained from Meteotrentino [28].

### Mosquito colony

We established an *Ae. koreicus* colony in the laboratory from larvae and pupae collected in the field (Castel Ivano, Italy; 46°02′N, 11°32′E; 360 m a.s.l) and kept in a climatic chamber at 23 ± 1 °C and a relative humidity of 75 ± 5%, with a photoperiod of 16L:8D with 1 h of dawn and 1 h of dusk [29]. Larvae were reared in 500 ml plastic cups filled with 250 ml of dechlorinated water and fed daily with finely ground cat food (Cat food Adult Fit 32TM, Royal Canin®, Aimargues, France). Adults were kept in a cage 45 × 45 × 45 cm (Bugdorm, MegaView Science Co., Ltd, Taiwan) supplied with cotton soaked in 10% sugar solution *ad libitum*. As suggested in Watson et al. [30], we set a black fabric on the back side of the cage to offer shaded oviposition and resting sites. Twice a week the colony was fed on cow blood for 1 h provided with Hemotek blood-feeding system (Hemotek Ltd., Accrington, England). Two ovitraps were placed inside the cage with filter paper as oviposition substrate. Strips with eggs were then removed and placed in plastic cups, stored in plastic bags loosely sealed and sprayed with water every week to maintain high RH [29].

### Egg-hatching experiment

One/two months-old colony eggs were placed in groups of 20 in white plastic glasses filled with 100 ml of dechlorinated water. Preliminary laboratory experiments showed that most of the newly laid eggs needed several days before being completely embryonated, thus we decided to use eggs old enough to be viable. Twenty replicates of 20 eggs each were made for each tested temperature, namely 8, 13, 23, 28 and 33 °C. Experiments at 13 °C and 23 °C were performed first; because survival did not show any remarkable difference (see “Results” section), we decided not to perform the experiment at intermediate temperatures. Five repetitions of 20 eggs each were used as controls at 23 °C for each temperature tested. RH and light were set as in the mosquito colony rearing conditions. Four mg of finely ground cat food was added to the water as hatching stimulus. The 20-eggs papers were left in water for 5 days and the number of larvae counted daily and removed.

### Larval survival and developmental time

A group of 10 less than 12-h-old first-instar larvae were placed in a plastic cup filled with 100 ml of dechlorinated water. We set 12 cups for each tested temperature, namely 4, 13, 18, 23, 28 and 33 °C. RH and light were set at the same values as mosquito colony rearing conditions. Every day exuviae and dead larvae were removed. The stage of each living larva was identified, and the number of each instar counted. Finely ground cat food was added daily to the water according to the larval stage: 0.1 mg/larva for L_1_; 0.2 mg/larva for L_2_; 0.3 mg/larva for L_3_; and 0.4 mg/larva for L_4_. The food amount was chosen consistently with the protocol proposed in [11], where *Ae. albopictus* larvae were fed daily, with the quantity of food increasing according to the stage of development (0.2 mg for L_1_, 0.4 mg for L_2_, 0.6 mg for L_3_, and 0.8 mg for L_4_). Preliminary experiments showed that halved quantities were still sufficient for *Ae. koreicus* larval development, as usually the placed food was not wholly consumed within one day. Every 48 h, excess food was removed, and half of the water was changed to avoid formation of bacterial scum. Cups were refilled with dechlorinated water to maintain the initial volume, and randomly rotated on the tray to prevent any position effects. Pupae were removed from the cups and isolated, and then the number of males and females was recorded.

### Adult longevity and gonotrophic cycle

Larvae were reared in 500 ml plastic cups filled with 250 ml of dechlorinated water in a climatic chamber at 23 ± 1 °C and 75 ± 5% RH with a photoperiod of 16L:8D with 1 h of dawn and 1 h of dusk, adapting the protocol adopted in [11]. Finely ground cat food was added daily to the water according to the larval stage: 0.1 mg/larva for L_1_; 0.2 mg/larva for L_2_; 0.3 mg/larva for L_3_; and 0.4 mg/larva for L_4_. Every 48 h, food excess was removed, and half of the water was changed to avoid formation of bacterial scum. Cups were refilled with dechlorinated water to maintain the initial volume. Pupae were isolated in plastic cups filled with 250 ml of dechlorinated water and placed in 22 × 22 × 22 cm cages (Bugdorm, MegaView Science Co., Ltd, Taiwan). Every day the cup with pupae was moved in another cage with the same size, therefore all the adults inside a cage were at the same age (˂ 24 h). Male and female mosquitoes were left together for 5 days for free mating and supplied with cotton soaked in 10% sugar solution *ad libitum*. After this period, females were allowed to feed on cow blood provided *via* a Hemotek blood-feeding system (Hemotek Ltd., Accrington, UK) for 30 min twice a week. Then, one male and one engorged female of the same age were isolated as a couple in a cage with an ovitrap lined with filter paper as oviposition substrate and 10% sugar solution *ad libitum*. We set 15 cages for the trials at 18, 28 and 33 °C, and 22 cages for the trial at 23 °C. To stimulate oviposition, the ovitrap was filled with 200 ml dechlorinated water and 5 ml of grass infusion prepared following the protocol proposed in [31]. A black fabric was set on the back side of the cage to offer shaded oviposition and resting sites. The date of blood meal and oviposition was recorded to calculate the gonotrophic cycle length of females. Ovitraps were checked daily for eggs laid on filter paper and, if present, counted and removed. The dates of death were recorded to calculate adult longevity.

### Temperature-dependent functions

For each immature stage, we computed daily death rates from measures of the average developmental time and survival. Taking as example the transition from eggs (*E*) to first-instar larvae (*L*_*1*_), we can describe the dynamics with the following system of ordinary differential equations (ODE)

$$E^{\prime} = - \left( {\tau_{E} + \mu_{E} } \right)E$$

$$L_{1}^{'} = \tau_{E} E$$

The experiment starts with *E*_*0*_ initial eggs that eventually develop into *k* (*k *≤ E_0_) first-instar larvae over an average time of 1/τ_E_ days. Thus, a simple calculation leads to compute the death rate as

$$\mu_{E} = \tau_{E} \left( {\frac{{E_{0} }}{k} - 1} \right).$$

Similarly, we computed $\mu_{j} \forall j \in \left\{ {L_{1} ,L_{2} ,L_{3} ,L_{4} ,P} \right\}$.

Subsequently, we modeled the mortality and developmental rates across different mosquito life stages as functions of temperature by fitting a suitable set of functions of the temperature *T*:

$$F_{1} \left( T \right) = \frac{{\exp \left( {a - b \cdot T} \right)}}{{1 + \exp \left( {a - b \cdot T} \right)}},\;F_{2} \left( T \right) = \frac{1}{{a + b \cdot { \exp }\left( {c \cdot T} \right)}},\;F_{3} \left( T \right) = a + b \cdot { \exp }\left( {c \cdot T} \right).$$

Uncertainty in model parameters was estimated by perturbing the computed functions with an additive error sampled from a normal distribution with variance equal to the average of the interpolation residuals [18].

### Model design

We modelled the population dynamics of *Ae. koreicus* by accounting for its 7 life stages, namely eggs (*E*), the four larval instars (*L*_*1*_, *L*_*2*_, *L*_*3*_, *L*_*4*_), pupae (*P*) and female adults (*A*). The model can be described with the following system of ODE:

$$E^{\prime} = n_{E} \tau_{A} A - \left( {\tau_{E} + \mu_{E} } \right)E$$

$$L_{1}^{'} = \tau_{E} E - \left( {\tau_{{L_{1} }} + \mu_{{L_{1} }} \left( {1 + \frac{L}{K}} \right)} \right)L_{1}$$

$$L_{2}^{'} = \tau_{{L_{1} }} L_{1} - \left( {\tau_{{L_{2} }} + \mu_{{L_{2} }} \left( {1 + \frac{L}{K}} \right)} \right)L_{2}$$

$$L_{3}^{'} = \tau_{{L_{2} }} L_{2} - \left( {\tau_{{L_{3} }} + \mu_{{L_{3} }} \left( {1 + \frac{L}{K}} \right)} \right)L_{3}$$

$$L_{4}^{'} = \tau_{{L_{3} }} L_{3} - \left( {\tau_{{L_{4} }} + \mu_{{L_{4} }} \left( {1 + \frac{L}{K}} \right)} \right)L_{4}$$

$$P^{\prime} = \tau_{{L_{4} }} L_{4} - \left( {\tau_{P} + \mu_{P} } \right)P$$

$$A^{\prime} = \frac{1}{2}\tau_{P} P - \left( {\mu_{A} + \chi \alpha \tau_{A} } \right)A$$

$$C^{\prime} = \chi \alpha \tau_{A} A$$

where τ_j_ and μ_j_ are respectively the temperature-dependent developmental and daily death rates of stage *j* with $j \in \left\{ {E,L_{1} ,L_{2} ,L_{3} ,L_{4} ,P,A} \right\}$. Similarly to what proposed for *Ae. albopictus*, mortality rates for immature stages and adults were multiplied by the same scaling factors as in [18] to take into account the lower survival in the field compared to laboratory-controlled conditions. The developmental rates correspond to egg hatching (τ_E_), larval molting (τ_L1_, τ_L2_, τ_L3_), pupation (τ_L4_), adult emergence (τ_P_) and gonotrophic cycle (τ_A_). *K* is a density-dependent scaling factor driving the carrying capacity for the larval stages (*L *= *L*_*1*_ + *L*_*2*_ + *L* _*3*_+ *L*_*4*_). The average number of eggs laid in one oviposition, n_E_, was set to 100 [32]. Female adults are trapped with rate α∙χ, where α is the daily capture rate of the traps and χ is a function of time defined equal to 1 when the trap is open and 0 otherwise. Since BG traps capture host-seeking mosquitoes, only a fraction A·τ_A_ of adults can be trapped. *C* represents the cumulative number of captured female adult mosquitoes. As only female adult mosquitoes are explicitly considered in the model, the term 1/2 in the equation for adults accounts for the sex ratio. The seasonal dynamics of the mosquito population was simulated from April 1 to October 31 for each site and year under study. Since no data are available on the overwintering mechanisms of *Ae. koreicus*, we simulated each year separately by initializing the system with 500 eggs.

The model has two free parameters: the daily capture rate of adult mosquitoes α and a larval density dependent factor *K*. While α is assumed to be equal among different traps and years, *K* is assumed to be both site- and year-specific. Thus, there are 13 parameters to estimate which form the set of unknown parameters Ψ. Specifically, $\varPsi = \left\{ {\alpha ,K\left( {s,y} \right)_{{s \in \left\{ {A,B,C,D} \right\},y \in \left\{ {2016,2017,2018} \right\}}} } \right\}$. The posterior distributions of Ψ were estimated by Markov Chain Monte Carlo (MCMC) sampling. The Poisson likelihood of the observed weekly captures given model-predicted ones was multiplied across the 12 datasets (4 traps and 3 years) under study to provide the overall likelihood of observations:

$$L = \mathop \prod \limits_{y = 2016}^{2018} \mathop \prod \limits_{{s \in \left\{ {A, \ldots ,D} \right\}}} \mathop \prod \limits_{m = 1}^{{M\left( {s,y} \right)}} e^{{ - C\left( {s,y,m,\psi } \right)}} \cdot \frac{{C\left( {s,y,m,\uppsi} \right)^{{n\left( {s,m,y} \right)}} }}{{n\left( {s,m,y} \right)!}}$$

where *y*, *s* and *m* run over the considered years, trapping sites and trapping sessions respectively, *M*(*s,y*) is the total number of trapping sessions carried out for the specific site and year, *n*(*s,m,y*) is the observed number of trapped adults and *C*(*s,y,m*,Ψ) is the number of captures predicted by the model with parameters Ψ. The posterior distribution of Ψ was obtained by using random-walk Metropolis-Hastings as an acceptance criterion, recursive normal jumps for parameter sampling and uninformative (uniform) priors.

We then applied the model to assess the influence of temperature on the population dynamics, using temperature records from the closest weather station to each trap site. To this aim, we re-simulated the mosquito abundance for each year *y* and site *s* using the estimated posterior distributions of Ψ and 10 different temperature patterns *T*_*s,y*_*(t)* ranging from $\overline{{T_{s,y} }} \left( t \right) - 2.5^\circ$ to $\overline{{T_{s,y} }} \left( t \right) + 2.5^\circ$, where $\overline{{T_{s,y} }} \left( t \right)$ is the recorded daily average temperature associated to year *y* and study site *s*.

Finally, we investigated whether *Ae. albopictus* is better adapted to the climate conditions of the study area by running the population model proposed in [18] for this species with the estimated posterior distributions of Ψ. In particular, we used the same capture rate α since our estimate was consistent (see “Results” section) with a previously published measure for this parameter for *Ae. albopictus* [33].

## Results

### Entomological collections

The total numbers of trapped female *Ae. koreicus* over all sites were 100, 145 and 214 in 2016, 2017 and 2018 respectively. A larger number of captures was observed in rural sites (C and D), which accounted for 36% and 26% of total collections, respectively. Capture data are presented in Additional file 1: Table S1.

### Laboratory experiments

**Fig. 2:**
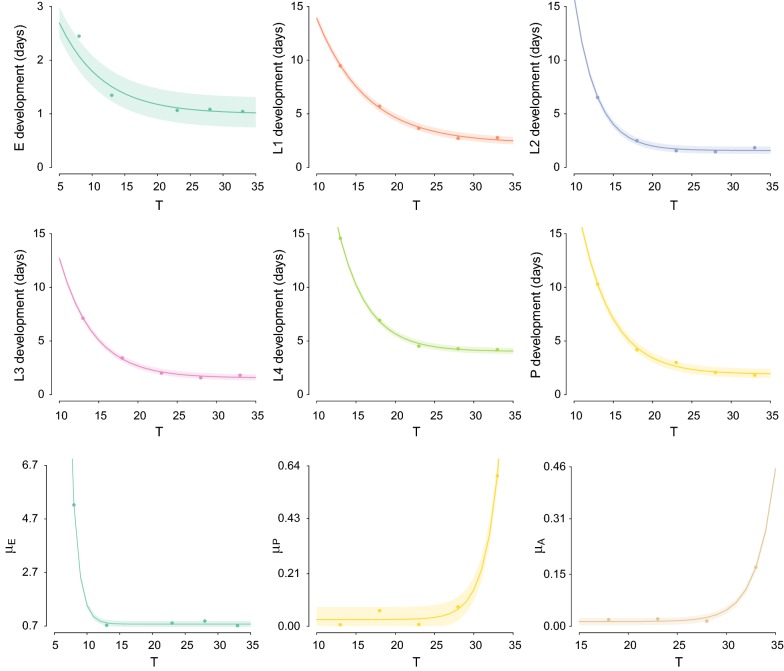
Temperature-dependent functions. Dots represent the experiments observations; shaded area represents 95% CI of predicted values

As shown in Fig. 2, higher temperatures shortened the developmental periods, with no substantial differences above 23 °C. Egg hatching rate was low at 8 °C, while it ranged between 50.50% and 52.25% for the other considered temperatures. At 4 °C, no first-instar larva survived. On the other hand, above 13 °C larval survival was quite high and seems little affected by temperature changes. Pupal and adult mortality increased considerably above 28 °C. In fact, adult females survived on average less than 6 days at 33 °C and at such temperature they never completed the gonotrophic cycle. Conversely, at the lower studied temperatures, females could survive for more than one month, taking on average between 9 and 15 days to complete the gonotrophic cycle. Observed survival rates, developmental times and longevities are reported in Tables 1, 2, 3.

**Table 1:** Experimental results for immature stages. Egg hatching rate (%) and larvae and pupae survival (%) at different temperatures obtained with the laboratory experiments. Numbers in parentheses represent the 95% CI

Stage	4 °C	8 °C	13 °C	18 °C	23 °C	28 °C	33 °C
E	–^a^	7.25 (4.70–9.80)	50.50 (45.60–55.40)	–^a^	53.75 (48.87–58.63)	51.00 (46.1–55.9)	57.25 (52.41–62.09)
L_1_	0	–^a^	95.00 (91.1–98.9)	90.83 (85.67–96.00)	80.00 (72.84–87.16)	80.83 (73.79–87.88)	81.67 (74.74–88.59)
L_2_	–^b^	–^a^	100	100	100	100	100
L_3_	–^b^	–^a^	100	99.08 (97.29–100)	100	98.97 (96.96–100)	95.92 (92.00–99.84)
L_4_	–^b^	–^a^	100	98.15 (95.61–100)	100	97.92 (95.06–100)	97.87 (94.96–100)
P	–^b^	–^a^	93.86 (89.31–98.41)	79.25 (70.57–87.92)	97.92 (95.03–100)	86.17 (78.65–93.69)	47.83 (33.07–62.59)

^a^The experiment was not performed

^b^All larvae died before completing the first molt

**Table 2:** Experimental results for immature stages: developmental time

Stage	8 °C	13 °C	18 °C	23 °C	28 °C	33 °C
E	2.45 (1.92–2.97)	1.35 (1.28–1.42)	–^a^	1.07 (1.03–1.10)	1.08 (1.04–1.12)	1.04 (1.01–1.07)
L_1_	–^a^	9.47 (9.15–9.80)	5.71 (5.51–5.90)	3.66 (3.49–3.83)	2.72 (2.60–2.84)	2.78 (2.64–2.92)
L_2_	–^a^	6.53 (6.34–6.71)	2.49 (2.31–2.67)	1.56 (1.46–1.66)	1.47 (1.36–1.58)	1.84 (1.71–1.98)
L_3_	–^a^	7.12 (6.96–7.28)	3.42 (3.10–3.73)	2.01 (1.91–2.11)	1.57 (1.41–1.73)	1.80 (1.66–1.95)
L_4_	–^a^	14.58 (14.30–14.85)	6.93 (6.76–7.11)	4.52 (4.31–4.73)	4.27 (4.07–4.48)	4.19 (3.99–4.38)
P	–^a^	10.31 (10.22–10.40)	4.19 (4.10–4.28)	3.00 (2.93–3.07)	2.07 (2.01–2.14)	1.82 (1.70–1.94)

^a^The experiment was not performed

*Notes*: Eggs (E): average time (days) between water immersion and hatching response. Larvae (L_1_-L_4_) and pupae (P): average developmental time (days). Numbers in parentheses represent the 95% CI

**Table 3:** Experimental results for female adults. Average female adult longevity (μ_A_^−1^) and gonotrophic cycle length (τ_A_^−1^) (days)

Rate	18 °C	23 °C	28 °C	33 °C
μ_A_^−1^	52.33 (29.06–75.61)	46.77 (30.55–62.99)	66.33 (58.30–74.37)	5.87 (4.6–7.14)
τ_A_^−1^	14.75 (3.10–26.40)	9.21 (7.12–11.30)	10.81 (7.55–14.06)	–^a^

^a^All females died before completing the gonotrophic cycle

*Note*: Numbers in parentheses represent the 95% CI

### Transition and death rates

All transition rates τ were fitted with logistic or exponential type functions (see Tables 4 and 5 and Fig. 2). The proposed functions interpolated quite well the experimental measures. In fact, the 95% confidence intervals (CI) of the predicted values were usually very narrow and the observed values always lie within them (Fig. 2). As temperature seems to have little influence on larval survival between 13 °C and 33°C, we decided to assume a temperature-independent daily death rate for each larval instar, obtained by averaging the rates observed at the different temperatures. Similarly, we assumed the gonotrophic cycle to last 11.6 days, the average computed with the experiments between 18 °C and 28 °C. As shown in Tables 4 and 5, the residuals between such averages and the estimates at different temperatures are quite small and comparable to those obtained with the fitted functions for the other rates.

**Table 4:** Temperature-dependent functions for transition rates τ_j_, $j \in \left\{ {E,L_{1} ,L_{2} ,L_{3} ,L_{4} ,P,A} \right\}$. Parameters for the temperature dependent functions for the development rates

Stage	Function	a	b	c	σ^2^
E	F_1_	0.15	1.28	–	0.033
L_1_	F_2_	2.26	56.31	− 0.16	0.025
L_2_	F_2_	1.59	496.08	− 0.35	0.023
L_3_	F_2_	1.56	111.14	− 0.23	0.016
L_4_	F_2_	4.03	337.00	− 0.27	0.018
P	F_2_	1.91	200.86	− 0.24	0.039
A	0.086^a^	–	–	–	4·10^−4^

^a^Constant value

*Note*: T denotes the temperature (°C); σ^2^ is the average of the residuals of the interpolation (see main text for additional details)

**Table 5:** Temperature-dependent functions for daily death rates µ_j_, $j \in \left\{ {E,L_{1} ,L_{2} ,L_{3} ,L_{4} ,P,A} \right\}$. Parameters for the temperature dependent functions for the daily death rates

Stage	Function	a	b	c	σ^2^
E	F_3_	0.77	7308.51	− 0.93	5·10^−3^
L_1_	0.05^a^	–	–	–	1.4·10^−3^
L_2_	0^a^	–	–	–	0
L_3_	0.007^a^	–	–	–	1·10^−4^
L_4_	0.003^a^	–	–	–	1∙10^−5^
P	F_3_	0.03	1.7·10^−8^	0.53	4·10^−4^
A	F_3_	0.01	5.6·10^−9^	0.52	4·10^−5^

^a^Constant value

*Note*: T denotes the temperature (°C); σ^2^ is the average of the residuals of the interpolation (see main text for additional details)

### Modelling results

**Fig. 3:**
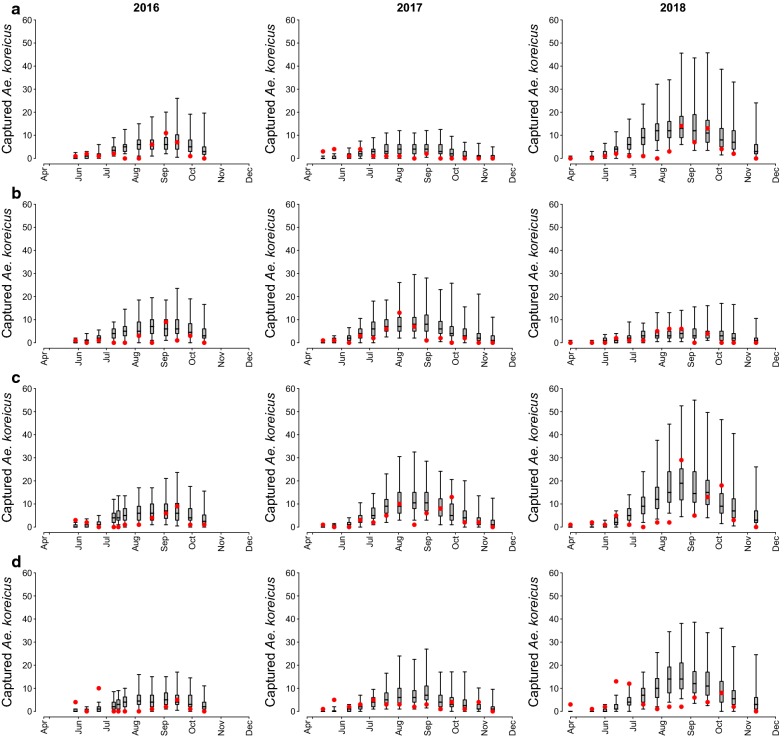
Model fit for each trapping site A, B, C, D (from first to last row) from 2016 to 2018 (from first to last column). Dots: recorded captures; boxplots (median, quartiles and 95% quantiles): predicted captures

As shown in Fig. 3, the mosquito population model fits the observed weekly captures quite well. Considering the 12 datasets (4 traps and 3 years), 82.3% of the observed captures lie within the 95% credible intervals of model predictions.

The estimated capture rate α was on average 0.157 (95% CI: 0.094–0.203), meaning that about 16% of the host-seeking females are captured in one day. Thus, an active trap might attract on average about 1.35% (α·τ_A_) of the adult females living in the trapped area. A similar percentage (1.64%) was estimated for *Ae. albopictus* with the same trap type in the same study region [33]. As expected, the larval density dependent factor *K* varied between sites and each site showed different values across the three years (see Additional file 2: Text S1). A strong positive correlation was found between *K*(*s*, *y*) and the total number of *Ae. koreicus* collected at site *s* during year *y* (Pearsonʼs correlation coefficient *r*_(10)_ = 0.96, P < 0.0001).

**Fig. 4:**
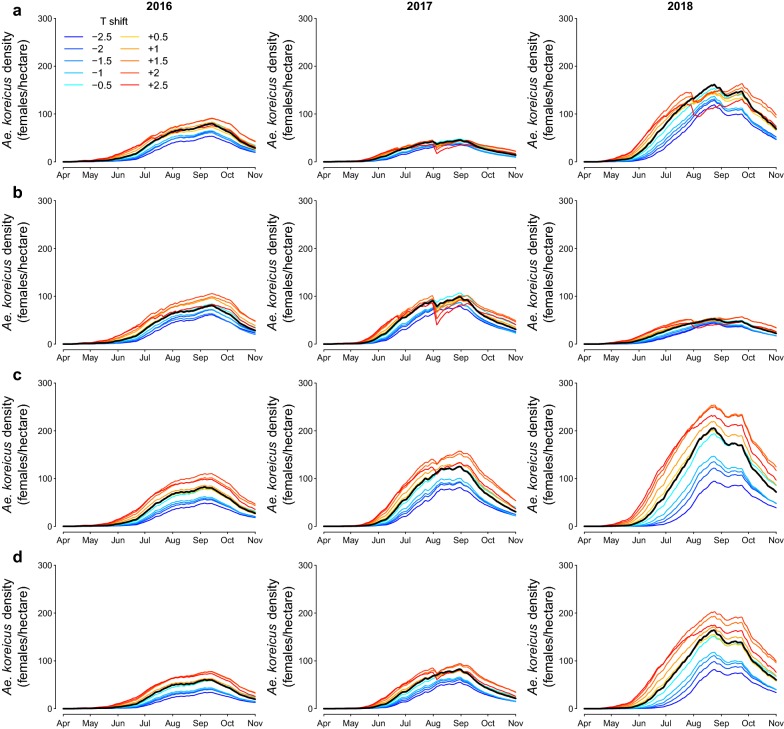
Effect of temperature on *Aedes koreicus*. Predicted average densities (number of adult females per hectare) for 10 different scenarios obtained varying daily temperature T from −2.5 °C (dark blue) to 2.5 °C (dark red) with a step of 0.5 °C for each trapping site A, B, C, D (from first to last row) and year (from first to last column). Black lines represent the estimated average abundance with no temperature perturbations

Figure 4 shows that changes in the daily average temperatures produced proportional effects on *Ae. koreicus* adult abundance from June onwards. Colder seasons are associated with a smaller size of adult populations, whereas hotter daily temperatures usually result in larger abundances and earlier occurrence of substantial densities. However, temperature peaks might cause temporary population drops (e.g. in all sites in 2017) and breeding seasons with sustained hot temperatures may be suboptimal for the maximal abundance during August and September (e.g. site A in 2018). Shifts in the daily temperatures may produce larger differences in specific some sites and years (e.g. site C in 2018).

**Fig. 5:**
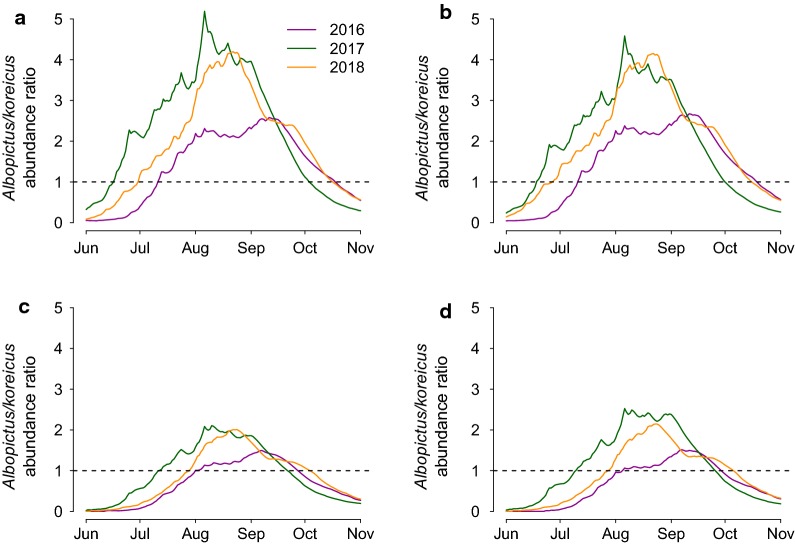
*Aedes albopictus/Ae. koreicus* ratio. Ratio between the average predicted adult *Ae. albopictus* and *Ae. koreicus* abundances for each trapping site A, B, C, D for each year under study

Figure 5 shows the predicted average ratio in adult abundance between *Ae. albopictus* and *Ae. koreicus*. Higher *Ae. albopictus* abundances are expected during the summer, while *Ae. koreicus* is more abundant at the beginning and end of the breeding season. In fact, *Ae. albopictus* is generally more adapted to higher temperatures; for instance, pupal development is faster for this species above 25 °C (see Additional file 2: Text S1). The ratio of the two predicted population sizes reached its peak between August and September and was higher for more urban and warmer sites (A and B, see Additional file 2: Text S1) and during hotter seasons (2016 *vs* 2018). Finally, in colder sites and years the abundance ratio favors *Ae. albopictus* later in the season, meaning that cold temperatures are more optimal for *Ae. koreicus* for a longer part of the breeding season.

## Discussion

In this study, we provided for the first time important data on the influence of temperature on the bionomics and population dynamics of *Ae. koreicus*. Colder temperatures slow the development of immature stages and decrease egg survival, especially below 10 °C. On the other hand, temperatures above 28 °C are very unfavorable for pupae and adults as they increase the death rates and prevent females from completing the gonotrophic cycle. Thus, the optimal thermal range for this species lies between 23 °C and 28 °C, where mortality is low across all stages and the development of immature stages is rapid.

A population dynamics model incorporating data on the effect of temperature on the mosquito life-cycle explained the observed temporal and spatial variability of the population density quite well. The model slightly overestimates the mosquito abundance at the end of the breeding season, when mosquitoes are known to be less active and possibly less likely to be captured. This reduction in activity is usually triggered by endogenous factors such as lower temperatures and photoperiod [34]. In addition, *Ae. albopictus* mosquitoes start to lay more resistant eggs at the end of the breeding season [34], which hatch only during the following spring, thereby reducing the number of emerging adults. A similar behavior may hold also for *Ae. koreicus*, but because of the lack of available data we could not explicitly consider it in our model.

We found that warmer seasonal temperatures usually cause an upper shift of the adult abundance curve and an anticipation of the breeding season, although very high temperatures in hotter months may cause sudden population drops. Warmer conditions are more likely to occur in the study area in the future because of climate change; indeed, a recent study suggested that temperatures might increase by up to 4 °C by the end of the 21st century throughout the Alpine region [35].

Captures were usually higher in the colder sites, possibly because of a larger availability of breeding sites, which is reflected by larger estimated larval scaling factors. In addition, previous studies in the same region showed that forested areas seem suitable for *Ae. koreicus*, while *Ae. albopictus* prefers artificial ones [5, 36].

Our findings clearly indicate that *Ae. koreicus* is still less adapted to the study region’s climate than *Ae. albopictus*. Although *Ae. koreicus* develops faster than *Ae. albopictus* at colder temperatures, the latter is more adapted to warmer conditions similar to the ones observed during summer in the province of Trento (see Additional file 2: Text S1 for a comparison of temperature-dependent rates). Our modelling results confirm that, given the same availability of breeding sites, *Ae. koreicus* might be more abundant than *Ae. albopictus* during the colder months of the breeding season, i.e. May-June and October-November. A weak larval interspecific competition has been demonstrated under laboratory conditions between these two species, with a slight advantage for *Ae. albopictus* [29]; however, shared breeding sites are not very common [5, 26]. Ecological competition could be taken into account in the model as previously done with *Cx. pipiens* (Linnaeus, 1758) [37] to assess its occurrence in the field, and this mechanism, similarly to diapause, might contribute to reduce the predicted overabundance at specific times of the year as it would decrease larval survival; however, this was beyond the scope of this work, also because of the limited available data.

We modelled mosquito population dynamics by considering only average daily temperatures, similarly to previous studies [10, 18–22, 37]; nonetheless, temperature fluctuations within a day might have a significant impact on mosquito abundance [38] and should be considered. However, we did not have access to temperature data at a finer temporal resolution than the daily average, and experiments on developmental rates were carried out at constant temperatures.

*Aedes koreicus* has been shown to be a competent vector for chikungunya virus [9] and *Dirofilaria immitis* [8]. In order to estimate pathogen transmission risks from the observed abundance of this species [19], several important factors still need to be quantified. For instance, the feeding preference, i.e. the preferred hosts for a blood meal, of *Ae. koreicus* has not been evaluated yet. Larvae of this species were found far from human settlings, indicating that the species can complete its life-cycle feeding on animals other than humans [26]. Another key parameter is the flight range, which is essential to compute the density of mosquito populations from capture data. The adult population estimated by our model depends on the attraction basin of the traps and eventually on the mosquito flight range. Here, because of the lack of available data, we assumed that the flight range of *Ae. koreicus* is equal to that of *Ae. albopictus* (150 m, see [39]), which resulted in densities in the same order of magnitude as *Ae. albopictus* in the same region [19]. An experimental estimate of the *Ae. koreicus* dispersal is crucial to accurately estimate the adult density, which is in turn critical to evaluate epidemiological risks for vector-borne pathogens.

Future studies, such as an assessment of the potential future areas of colonization of this species or an estimate of the transmission risk of an *Ae. koreicus*-borne pathogen, will certainly benefit from the present results, including the entomological dataset (Additional file 1: Table S1). In particular, our temperature-dependent model might be used, in the future, in an epidemiological framework to estimate pathogen transmission risks [18, 19, 22].

## Conclusions

This study provides, to our knowledge, the first experimental data on how temperature affects the bionomics of *Ae. koreicus* mosquitoes, a new invasive mosquito species in Europe. The most favorable temperature range is between 23 °C and 28 °C, while hotter temperatures produce excessive pupal and adult mortality and block the gonotrophic cycle. These results explain the recent success of this species at establishing into temperate areas of Europe. Our modelling results validate these findings by reproducing population dynamics of the mosquito in the wild, predict the nonlinear effect of different temperature scenarios and highlight the need to investigate further the feeding and dispersal behavior of this species to estimate quantitatively the risks of transmission of pathogenic agents associated with *Ae. koreicus*.

## Supplementary information

**Additional file 1: Table S1.** Observed *Ae. koreicus* time series used for model calibration.

**Additional file 2: Text S1.** Supporting text containing methodological details and additional results.

## Data Availability

All data generated or analyzed during this study are included in this published article, its bibliography and its additional files.

## Abbreviations

RH: relative humidity
CI: confidence interval
