# Supplementary material for: First report of the influence of temperature on the bionomics and population dynamics of Aedes koreicus, a new invasive alien species in Europe
Source: Parasit Vectors. 2019 Nov 6;12:524. doi: 10.1186/s13071-019-3772-5 (PMC6833271; doi:10.1186/s13071-019-3772-5)
Supplement: Supplementary file 2 — Additional file 2: Text S1. Supporting text containing methodological details and additional results. [file 13071_2019_3772_MOESM2_ESM.pdf]

First report of the influence of temperature on the bionomics and population dynamics of *Aedes koreicus*, a new invasive alien species in Europe

Giovanni Marini[1,2], Daniele Arnoldi[1], Frederic Baldacchino[1,3], Gioia Capelli[4], Giorgio Guzzetta[2,5], Stefano Merler[2,5], Fabrizio Montarsi[4], Annapaola Rizzoli[1], Roberto Rosà[1,2,6]

1) Department of Biodiversity and Molecular Ecology, Research and Innovation Centre, Fondazione Edmund Mach, San Michele all'Adige (Trento), Italy;
2) Epilab-JRU, FEM-FBK Joint Research Unit, Province of Trento, Italy;
3) Direction départementale de la protection des population du Nord, Lille, France;
4) Laboratory of Parasitology, Istituto Zooprofilattico Sperimentale delle Venezie, Legnaro (PD), Italy;
5) Center for Information Technology, Bruno Kessler Foundation, Trento, Italy;
6) Center Agriculture Food Environment, University of Trento, San Michele all'Adige (TN), Italy.

Additional file 2 Text S1

Temperature trends

The climate of our study area is temperate-oceanic with three main areas: subcontinental (the main valleys with more severe winters), continental (the alpine valleys) and alpine (the areas above the treeline) [1]. Clearly (see also Table S1) sites A and B, associated to Trento station, are characterized by warmer conditions.

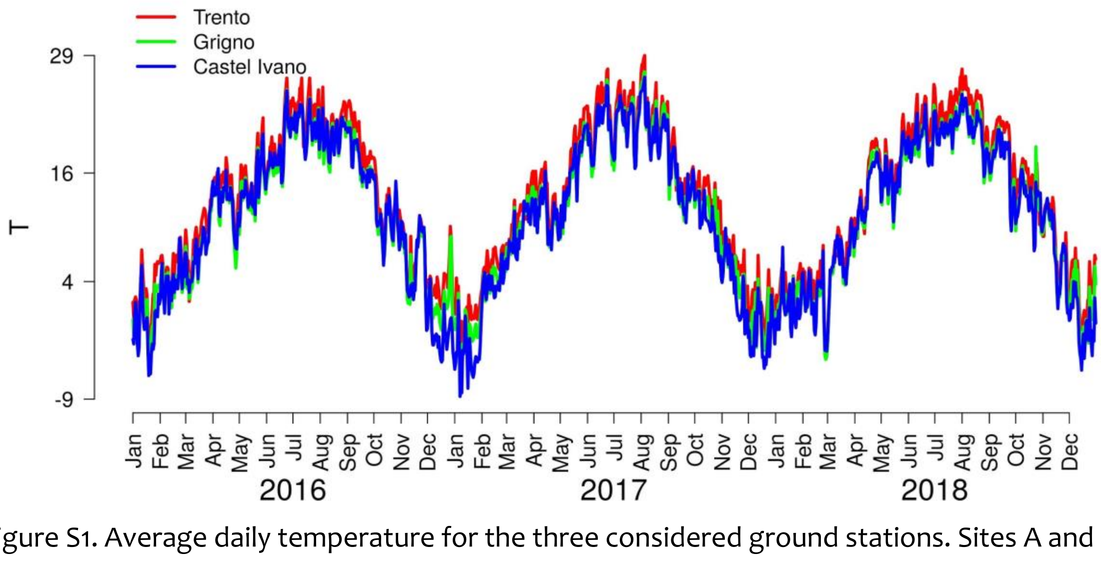

Figure S1. Average daily temperature for the three considered ground stations. Sites A and B are associated to Trento station (red line), C to Grigno station (green line) and D to Castel Ivano station (blue line).

Table S1. Study period (April-October) average temperature (°C).

Weather station	2016	2017	2018
**Trento**	17.9	18.4	19.1
**Grigno**	16.5	16.7	17.4
**Castel Ivano**	16.4	16.9	17.5

Ae. koreicus total captures

As shown in Figure S2, in sites C and D the number of trapped *Ae. koreicus* mosquitoes was higher with respect to the other two sites.

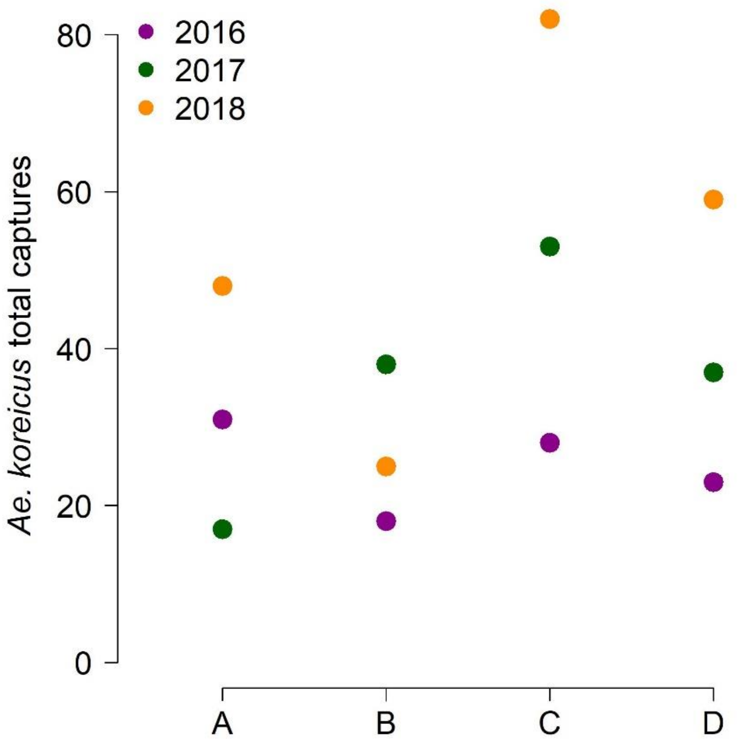

Figure S2. Number of captured *Ae. koreicus* female mosquitoes per site (A, …, D) and year.

Comparison between *Ae. koreicus* and *Ae. albopictus*

By comparing temperature-dependent developmental and survival rates computed for *Ae. koreicus* (this study) and *Ae. albopictus* [2] in Figure S3, we can note that *Ae. albopictus* is generally more adapted to higher temperatures. For instance, adult survival starts to increase

exponentially only after 35°C, whereas already at 33°C we observed a sharp reduction in *Ae. koreicus* longevity.

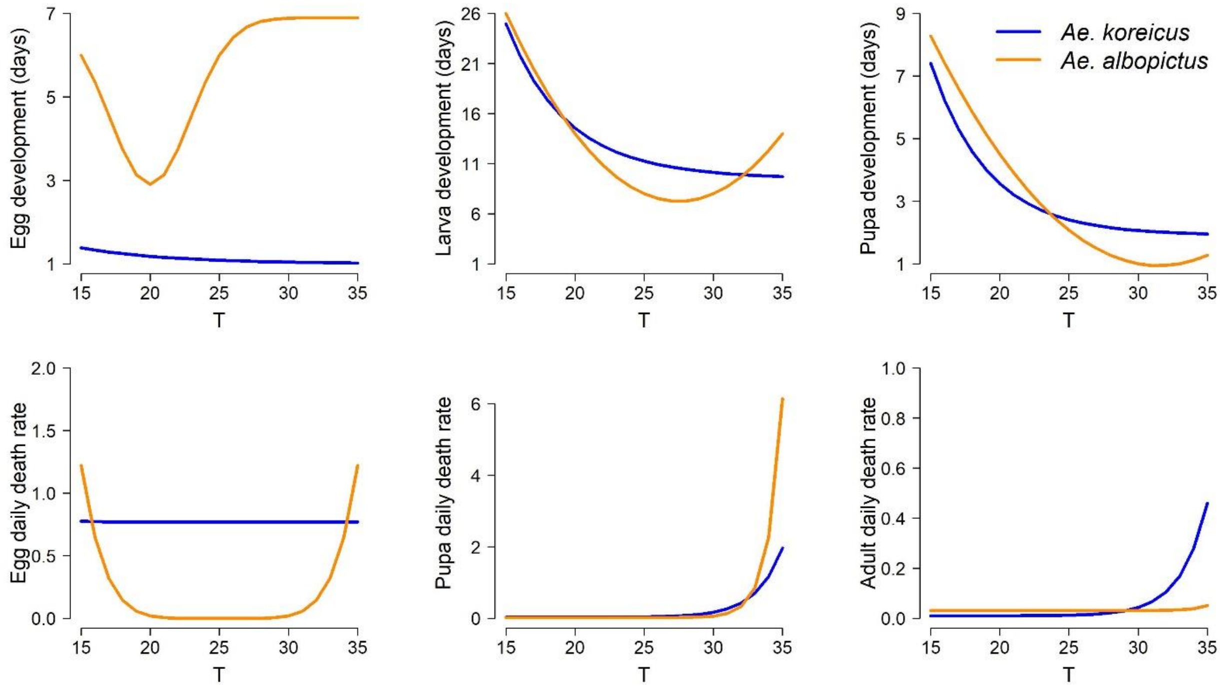

Figure S3. Comparison between *Ae. koreicus* (blue) and *Ae. albopictus* (orange) temperature-dependent developmental lengths (eggs, larvae and pupae, first row) and death rates (eggs, pupae and adults, second row).

Estimated distributions of model free parameters

Figure S4 reports the distributions of the model free parameters. Higher density dependent factors are associated with a greater number of captured mosquitoes. Figure S5 shows the relationship between the average of the estimated distribution of the density dependent factor $K(s, y)$ and total number of *Ae. koreicus* collected at site s during year y. As reported in the main text, such relationship is significantly positive.

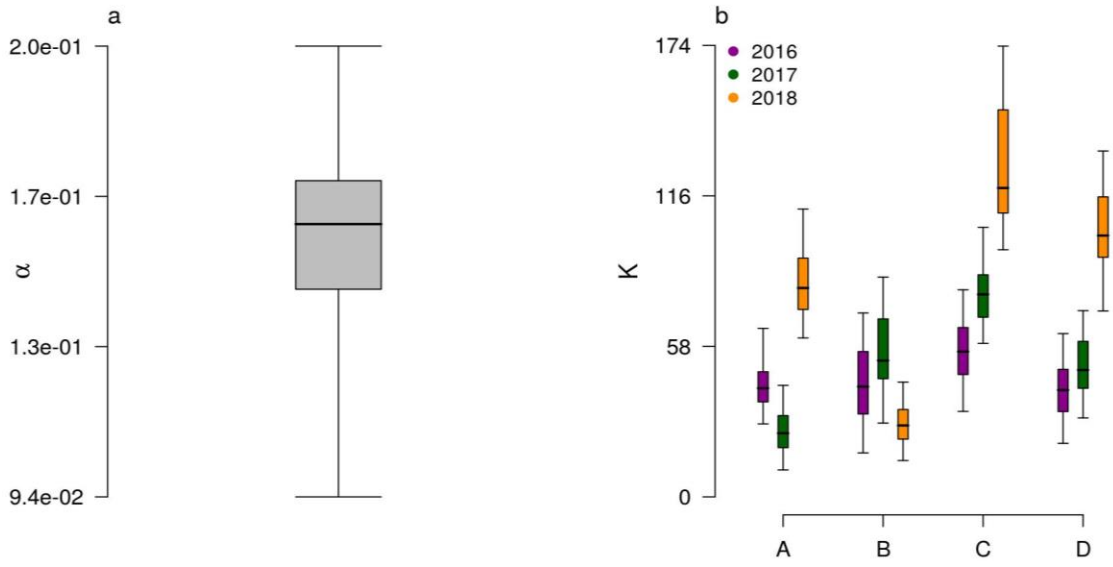

Figure S4. Boxplots (median, quartiles and 95% quantiles) of the estimated distributions of the model free parameters. a) α, the capture rate; b) *K*, the larval density dependent factor, which is year (2016, … , 2018) and site (A, … , D) dependent.

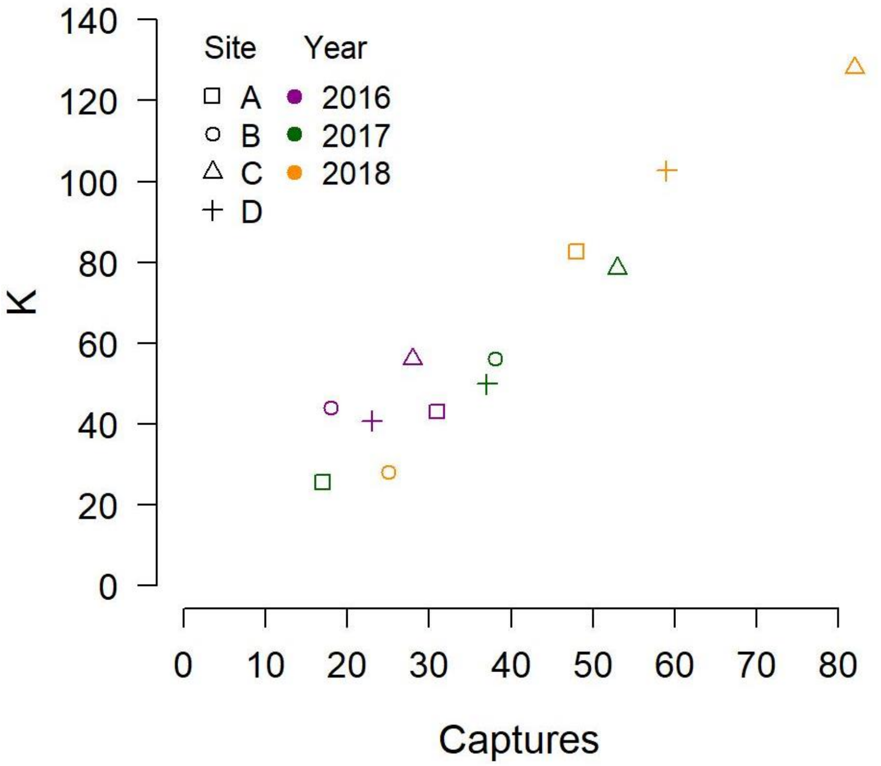

Figure S5. Relationship between the average density dependent factor *K* and the total number of trapped *Ae. koreicus* for each site (A, … , D) and year (2016, 2017, 2018) under study.

Estimated *Ae. koreicus* density

If we assume *Ae. koreicus* flight range r is 150m, similarly to what found for *Ae. albopictus* [3], then by dividing A(t), the adult mosquito abundance computed through our model, by $\pi \cdot r^2$, we

can estimate the mosquito density, i.e. the number of adult females per unit of space (e.g. per hectare), which is shown in Figure S6.

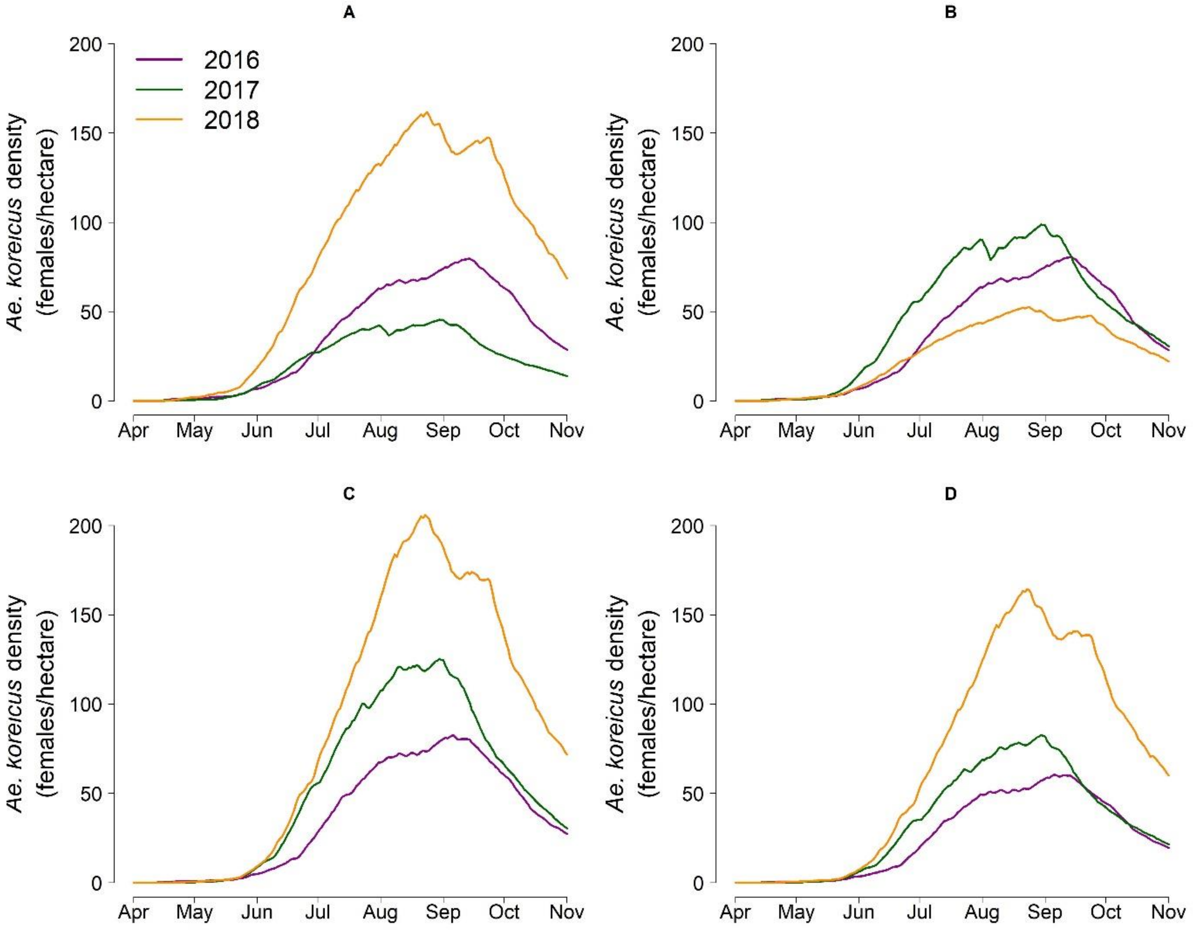

Figure S6. Estimated average number of *Ae. koreicus* adult females per hectare for each year (2016, …, 2018) and site (A, …, D) under study.

Bibliography

1. Neteler M, Roiz D, Rocchini D, Castellani C, Rizzoli A. Terra and Aqua satellites track tiger mosquito invasion: modelling the potential distribution of *Aedes albopictus* in north-eastern Italy. Int J Health Geogr. 2011; 10:49.
2. Poletti, P. et al. Transmission potential of chikungunya virus and control measures: the case of Italy. PLoS ONE. 2011; 6:e18860.
3. Marini F, Caputo B, Pombi M, Tarsitani G, Della Torre A. Study of *Aedes albopictus* dispersal in Rome, Italy, using sticky traps in mark-release-recapture experiments. Med Vet Entomol. 2010; 24(4):361–8.
